# Metabolic syndrome and the incidence of knee osteoarthritis: A meta-analysis of prospective cohort studies

**DOI:** 10.1371/journal.pone.0243576

**Published:** 2020-12-23

**Authors:** Daqing Nie, Guixin Yan, Wenyu Zhou, Zhengyi Wang, Guimei Yu, Di Liu, Na Yuan, Hongbo Li

**Affiliations:** 1 Department of Rheumatic Disease, The Affiliated Hospital of Changchun University of Chinese Medicine, Changchun, China; 2 The Innovation Practice Center, Changchun University of Chinese Medicine, Changchun, China; 3 Department of Immunology and Rheumatology, The Fourth Hospital of Wuhan, Wuhan, China; 4 College of Nursing and Health Science, Nanfang Medical College of Sun Yat-Sen University, Guangzhou, China; 5 Department of Rheumatic Disease, The Third Clinical Affiliated Hospital of Changchun University of Chinese Medicine, Changchun, China; Monash University, AUSTRALIA

## Abstract

**Background:**

Cross-sectional studies suggest an association between metabolic syndrome (MetS) and knee osteoarthritis (KOA). We performed a meta-analysis to evaluate whether MetS is an independent risk factor for KOA.

**Methods:**

Prospective cohort studies evaluating the association between MetS and KOA in general population were retrieved from PubMed and Embase. Only studies with multivariate analyses were included. Data were pooled with a random-effect model, which is considered to incorporate heterogeneity among the included studies.

**Results:**

Five studies including 94,965 participants were included, with 18,990 people with MetS (20.0%). With a mean follow-up duration of 14.5 years, 2,447 KOA cases occurred. Pooled results showed that MetS was not significant associated with an increased risk of KOA after controlling of factors including body mass index (adjusted risk ratio [RR]: 1.06, 95% CI: 0.92~1.23, p = 0.40; I^2^ = 33%). Subgroup analysis showed that MetS was independently associated with an increased risk of severe KOA that needed total knee arthroplasty (RR = 1.16, 95% CI: 1.03~1.30, p = 0.02), but not total symptomatic KOA (RR = 0.84, 95% CI: 0.65~1.08, p = 0.18). Stratified analyses suggested that MetS was independently associated with an increased risk of KOA in women (RR = 1.23, 95% CI: 1.03~1.47, p = 0.02), but not in men (RR = 0.90, 95% CI: 0.70~1.14, p = 0.37).

**Conclusions:**

Current evidence from prospective cohort studies did not support MetS was an independent risk factor of overall KOA in general population. However, MetS may be associated with an increased risk of severe KOA in general population, or overall KOA risk in women.

## Introduction

Metabolic syndrome (MetS) is a cluster of cardiometabolic abnormalities including abdominal adiposity, insulin resistance, hypertension, and dyslipidemia [1]. Accumulating evidence suggests that MetS is related to the pathogenesis of many chronic diseases, such as cardiovascular diseases [2] and cancer [3]. Osteoarthritis (OA) is the most common category of arthritis, which has become one of the leading causes of morbidity worldwide [4]. Accumulating evidence has indicated that both MetS and OA are related with aging [5,6].

Pathophysiologically, MetS is characterized of low-grade systematic inflammation [7], which may substantially contribute to the features of OA, such as subchondral bone remodeling and formation of osteophytes [8–10]. Accordingly, MetS has been hypothesized as a risk factor for the development of OA. Previous studies showed that patients with OA of the knee (KOA), the most frequently affected joint, have significantly higher prevalence of MetS and its components [11,12]. Moreover, subsequent observational studies suggest a strong association between MetS and KOA [8,13]. However, most of these studies are cross-sectional, and body mass index (BMI), a confirmed risk factor for KOA [14], was not adjusted [15]. Therefore, the sequential relationship between MetS and KOA remains undetermined. Moreover, it is also unknown whether the potential association between MetS and KOA remains after adjustment of BMI. Therefore, we aimed to perform a meta-analysis of prospective cohort studies to evaluate whether MetS is an independent risk factor for KOA.

## Methods

The meta-analysis was designed and performed in accordance with the Cochrane’s Handbook [16], and reported in accordance with the MOOSE (Meta-analysis of Observational Studies in Epidemiology) [17] guideline.

### Literature searching

Electronic databases of PubMed and Embase were systematically searched using the combination of the following terms: (1) metabolic syndrome" OR "insulin resistance syndrome" OR "syndrome X"; and (2) "osteoarthritis". We did not limit the study design and site of OA in this search strategy in order not to miss potential relevant studies. The search was limited to studies published in English. The reference lists of original and review articles were also analyzed manually. The final literature search was performed on April 14, 2020.

### Study selection

Studies were included if they met the following criteria: (1) published as full-length article in English; (2) designed as prospective cohort studies with a minimal follow-up duration of one year; (3) included general population without KOA at baseline; (4) participants with MetS were identified as exposure of interest at baseline; (5) participants without MetS at baseline were included as controls; (6) documented the incidence of KOA during follow-up; and (7) reported the adjusted risk ratios (RRs, at least adjusted for age and gender) and their corresponding 95% confidence intervals (CIs). For studies reporting adjusted RR in multiple models, the one with the most adequately adjusted model was used. Definitions of MetS were consistent with that was applied in the original studies. Reviews, editorials, preclinical studies, and non-cohort studies were excluded. Conference proceedings were not considered because these reports may not be strictly peer-reviewed, which confound the results of the meta-analysis.

### Data extracting and quality evaluation

Literature search, data extraction, and study quality assessment were independently performed by two authors according to the predefined inclusion criteria. If inconsistencies occurred, discussion with the corresponding author was indicated to resolve the disagreements. The following data were extracted: (1) name of the first author, publication year, study location; (2) characteristics and numbers of the participants, criteria for the diagnosis of MetS, number and prevalence of participants of MetS at baseline, and follow-up durations; and (3) definition of KOA outcome, number of KOA cases occurred during follow-up, and variables adjusted in the multivariate analysis. The quality of each study was evaluated using the Newcastle-Ottawa Scale [18]. This scale ranges from 1 to 9 stars and judges the quality of each study regarding three aspects: selection of the study groups; the comparability of the groups; and the ascertainment of the outcome of interest.

### Statistical analyses

The association between MetS and KOA incidence was measured by RRs in this study. To stabilize its variance and normalized the distribution, RR data and its corresponding stand error (SE) from each study was logarithmically transformed [16], as previously described [19]. The Cochrane’s Q test was performed to evaluate the heterogeneity among the include cohort studies [16,20], and the I^2^ statistic was also calculated. A significant heterogeneity was considered if I^2^ > 50%. A random effect model was used to pool the results since this model has been suggested to incorporate the potential heterogeneity among the included studies [16]. Sensitivity analyses, by omitting one study at a time, were performed to evaluate the potential influence of certain study on the outcome of the meta-analysis [21]. Subgroup analyses were performed to evaluate the influence of difference in KOA definitions among the include studies on the outcomes. Moreover, stratified analyses were performed to evaluate the potential gender-difference of the results [16]. Potential publication bias was assessed by visual inspection of the symmetry of the funnel plots, complemented with the Egger regression test [22]. The RevMan (Version 5.1; Cochrane Collaboration, Oxford, UK) and STATA software were used for the statistics.

## Results

### Literature search

**Fig 1:**
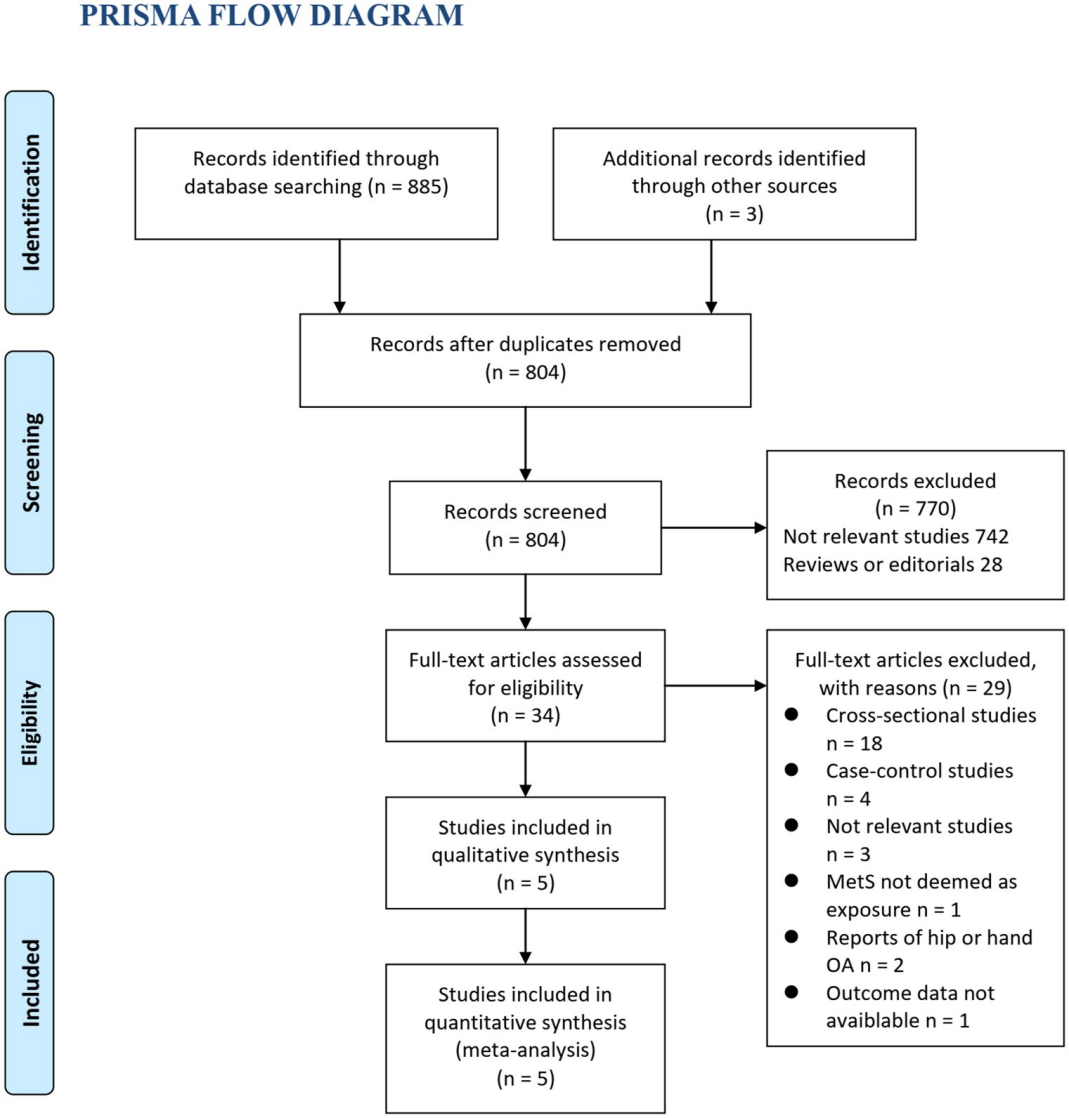
Flowchart of database search and study identification. *From*: Moher D, Liberati A, Tetzlaff J, Altman DG, The PRISMA Group (2009). *P*referred *R*eporting *I*tems for Systematic Reviews and *M*eta-*A*nalyses: The PRISMA Statement. PLoS Med 6(7): e1000097. doi:10.137l/journal.pmed1000097. **For more information, visit www.prisma-statement.org**.

Process of literature search is summarized in Fig 1. Briefly, 804 studies were obtained via initial database search after removing of duplication. Among them, 770 were excluded via screening of titles and abstracts, primarily because they were not relevant to the purpose of the meta-analysis. For the remaining 34 potential relevant studies that underwent full text review, 29 were further excluded based on reasons listed in Fig 1. Finally, five prospective cohort studies were included in the meta-analysis [23–27]. The three studies identified by manual search were not finally included in the meta-analysis because they were not relevant to the aim of the meta-analysis [28,29], or not a prospective cohort study [30].

### Study characteristics and quality evaluation

The characteristics of the included studies are summarized in Table 1. Overall, five prospective cohort studies with 94,965 community-derived participants were included in the meta-analysis [23–27]. These studies were published between 2009 and 2019, and performed in Sweden, Austria, the Unites States, Norway, and Finland, respectively. The mean ages of the included participants varied from 49 to 65 years, and the proportions of male ranged from 40% to50%. As for the diagnostic criteria for MetS, all of the included used the adult treatment panel III-national cholesterol education program (ATPIII-NCEP) criteria except for one study [23,25–27], in which the International Diabetes Federation (IDF) criteria was used [24]. According to the ATPIII-NECP criteria, subjects were considered to have MetS if they met at least 3 of the following 5 criteria: 1) abdominal obesity (waist circumference ≥ 102 cm in men and ≥ 88 cm in women), 2) high triglyceride levels (≥ 150 mg/dl), 3) low HDL cholesterol (< 40 mg/dl in men and < 50 mg/dl in women), 4) high blood pressure (systolic blood pressure ≥ 130 mmHg or diastolic blood pressure ≥ 85 mmHg or treatment for high blood pressure), and 5) high fasting glucose (≥ 110 mg/dl or diagnosis of diabetes) [31]. According to the IDF criteria, a person was defined to have MetS if he/she has central obesity (waist circumference ≥ 94 cm for men and ≥8 0 cm for women) and any two of the following four factors: raised serum triglyceride level (≥ 1.7 mmol/L), reduced serum HDL cholesterol level (< 1.03 mmol/L for males and <1.29 mmol/L for females, or specific treatment for these lipid abnormalities), raised blood pressure (systolic blood pressure ≥ 130 mmHg or diastolic blood pressure ≥ 85 mmHg, or treatment of previously diagnosed hypertension) and impaired fasting glycaemia (fasting plasma glucose > 5.6 mmol/L or previously diagnosed type 2 diabetes) [32]. Accordingly, 18,990 people had MetS (20.0%) at baseline. The follow-up durations varied from 6.8 to 32.0 years. As for the definitions of KOA outcome, three studies observed the incidence of severe KOA that needs total knee arthroplasty (TKA) [23,24,26], one study observed KOA related hospitalization [27], and another one evaluated the incidence of symptomatic KOA evidenced by radiography [25]. For the study by Niu et al. [25], subjects were considered to have symptomatic KOA if they had both knee pain (self-reported pain, aching or stiffness of either knee) and radiographic KOA in either tibiofemoral or patellofemoral joint on radiographs. The radiographic KOA in this study was diagnosed if the patient had a Kellgren/Lawrence grade of ≥ 2 on the anteroposterior view, or any osteophyte grade of ≥ 2, or any osteophyte grade of ≥ 1 plus a joint space narrowing grade of ≥ 2 in the patellofemoral joint on the lateral view [25]. With a mean follow-up duration of 14.5 years, 2,447 KOA cases occurred. All of the studies analyzed the association between MetS and incidence of KOA with multivariate analyses, with adequate adjustment of known risk factors for KOA, including age, sex, BMI, and level of physical activity. The qualities of the cohort studies were good, with the NOS ranging from eight to nine points (Table 2).

**Table 1:** Characteristics of the included prospective cohort studies.

Study	Country	Participants	Sample size	Mean age	Male	BMI	Definition of MetS	Prevalence of MetS at baseline	Follow-up duration	Definition of KOA and outcome collection	No. of KOA patients	Variables adjusted
				years	%	kg/m^2^		% (n =)	years			
Engstrom 2009 [23]	Sweden	General population	5171	57.5 ± 5.9	41.5	26.0	ATPIII-NCEP	22.2 (1148)	12.4	KOA patients undergoing TKA according to ICD-9/10 codes	89	Age, sex, BMI, smoking, CRP, and physical activity
Hussain 2014 [24]	Australia	General population	19868	64.8 ± 8.5	40.7	26.9	IDF	19.3 (3833)	6.8 ± 1.5	KOA patients undergoing TKA according to the National Joint Replacement Registry	660	Age, sex, BMI, country of birth, education, and physical activity
Niu 2017 [25]	the US	General population	991	54.2 ± 8.1	44.9	28.4	ATPIII-NCEP	24.6 (244)	10	Symptomatic KOA evidenced by self-reported symptom’s and radiography during the follow-up by physicians	128	Age, sex, BMI, education, smoking, alcohol consumption, and physical activity
Hellevik 2018 [26]	Norway	General population	62661	49.8 ± 16.9	47.4	NR	ATPIII-NCEP	20.1 (12593)	15.4 ± 4.3	KOA patients undergoing TKA according to the National Arthroplasty Registry	1111	Age, sex, smoking, BMI, physical activity, and education
Konstari 2019 [27]	Finland	General population	6274	49.9 ± 13.6	49.9	25.7	ATPIII-NCEP	18.7 (1172)	32	KOA related hospitalization according to ICD-9/10 codes	459	Age, sex, BMI, history of physical workload, smoking, knee complaint, and previous knee injury

KOA, knee osteoarthritis; MetS, metabolic syndrome; US, United States; ATPIII-NCEP, the adult treatment panel III-national cholesterol education program; IDF, International Diabetes Federation; TKA, total knee arthroplasty; BMI, body mass index; CRP, C-reactive protein; NR, not reported; ICD, International Classification of Diseases.

**Table 2:** Study quality evaluation by the Newcastle-Ottawa Scale.

Study	Representativeness of the exposed cohort	Selection of the non-exposed cohort	Ascertainment of exposure	Outcome not present at baseline	Control for age and sex	Control for other confounding factors	Assessment of outcome	Enough long follow-up duration	Adequacy of follow-up of cohorts	Total
Engstrom 2009	1	1	1	1	1	1	0	1	1	8
Hussain 2014	1	1	1	1	1	1	0	1	1	8
Niu 2017	1	1	1	1	1	1	1	1	1	9
Hellevik-2018	1	1	1	1	1	1	0	1	1	8
Konstari 2019	1	1	1	1	1	1	0	1	1	8

### Results of overall meta-analysis

**Fig 2:**
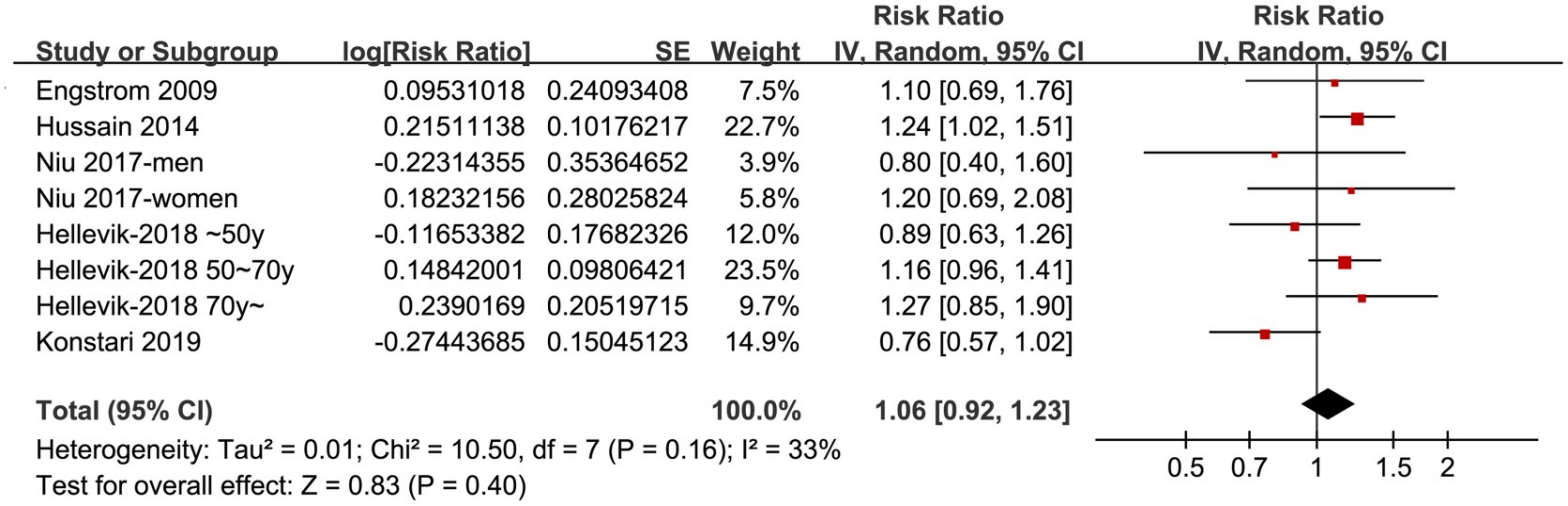
Forest plots for the meta-analysis of the association between MetS and KOA incidence in general population.

Since two of the included studies reported stratified data by sex (two datasets) [25] and age (three datasets) [26] of the participants rather than the results of the overall population, these datasets were included independently. Moderate heterogeneity was detected among the included studies (p for Cochrane’s Q test = 0.16, I^2^ = 33%). Pooled results with a random-effect model showed that MetS was not significant associated with an increased risk of overall KOA after controlling of confounding factors including age, sex, BMI, and physical activities et al (adjusted risk ratio [RR]: 1.06, 95% CI: 0.92~1.23, p = 0.40; Fig 2).

### Results of additional analysis

**Fig 3:**
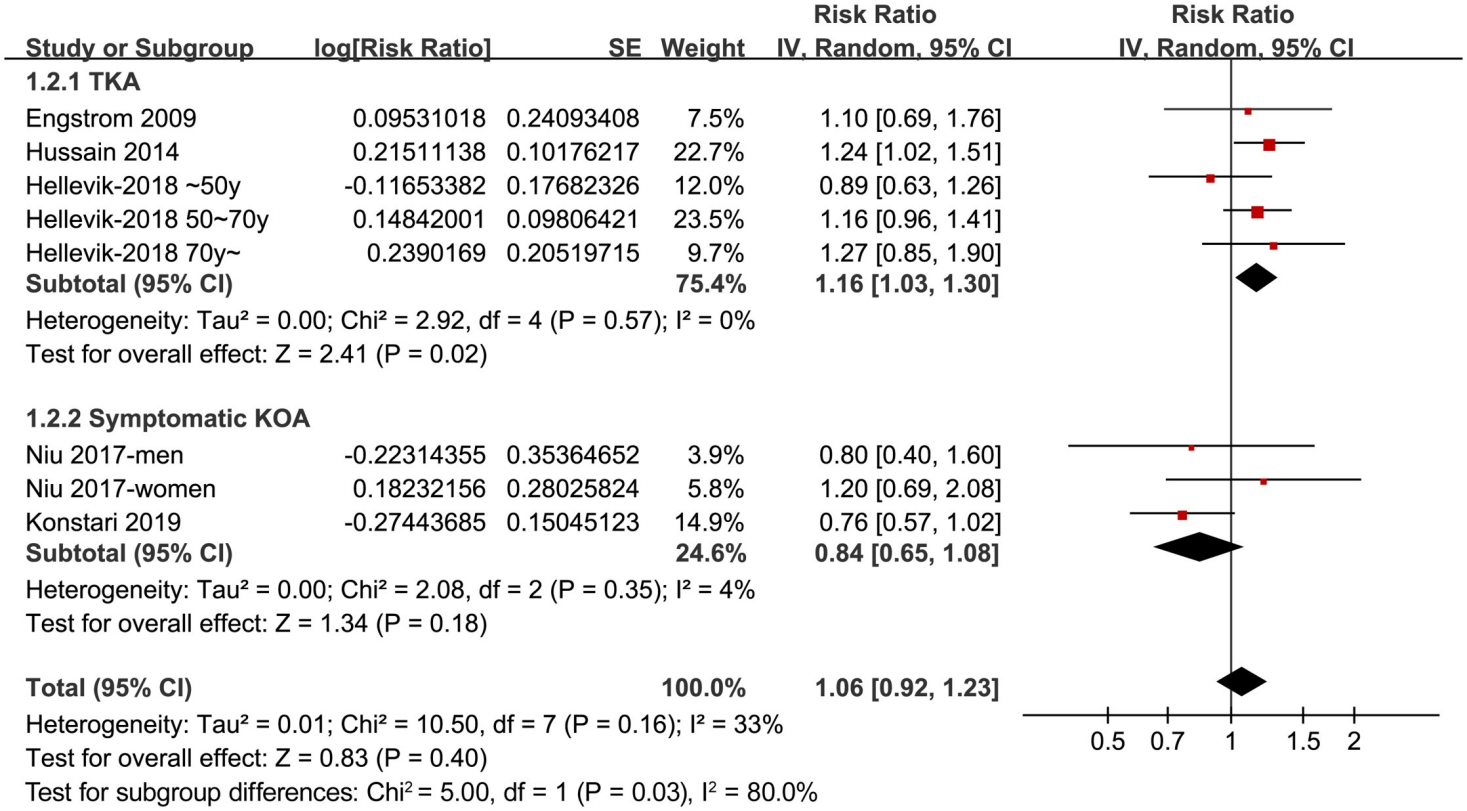
Forest plots for the subgroup analyses of the association between MetS KOA according to the definitions of KOA events.

**Fig 4:**
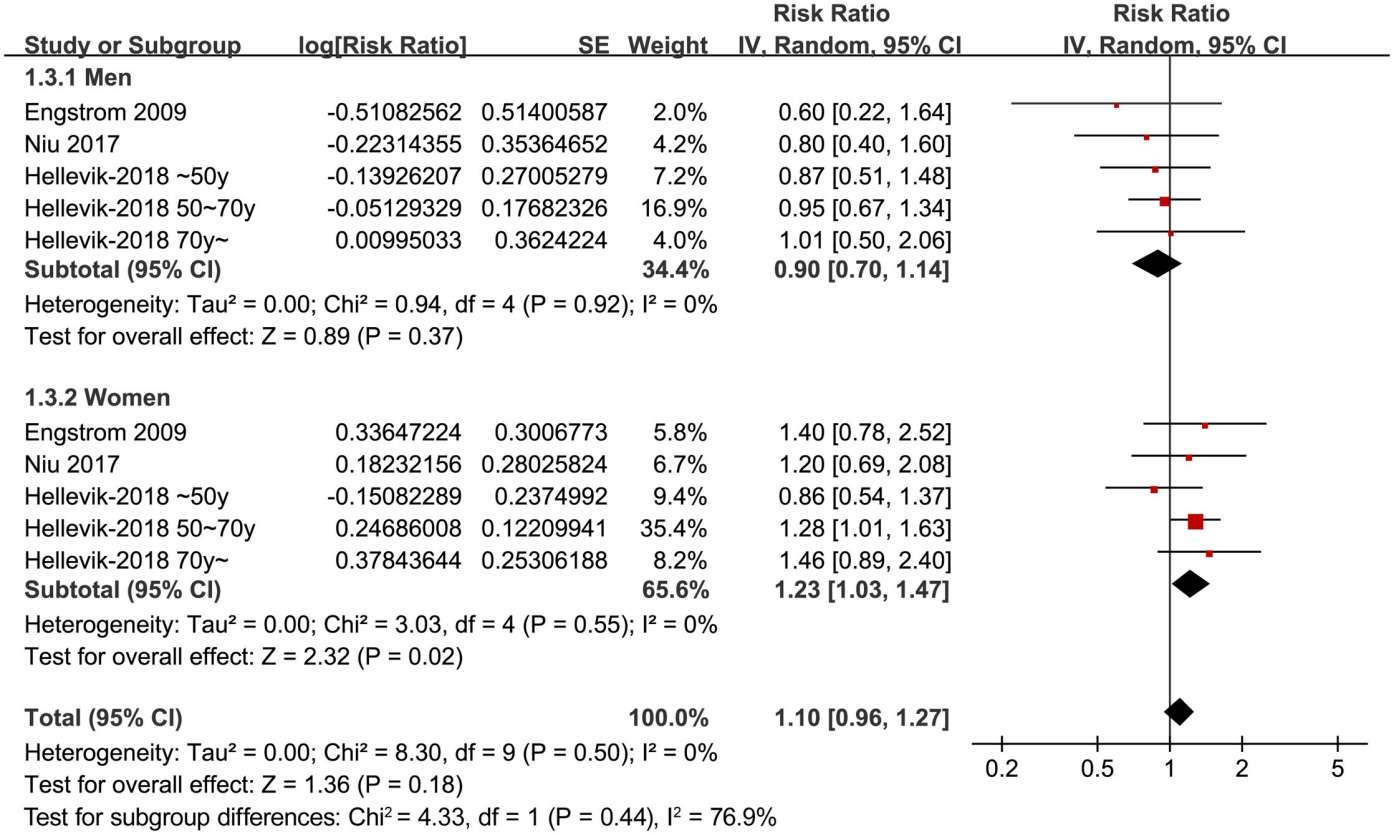
Forest plots for the stratified analyses of the association between MetS KOA according to the sex of the participants.

Sensitivity analysis by excluding the study which used IDF criteria for the diagnosis of MetS did not change the results (adjusted RR: 1.02, 95% CI: 0.87~1.19, p = 0.84; I^2^ = 26%). Moreover, excluding the study with the longest follow-up duration (32 years) [27] showed similar results (adjusted RR: 1.09, 95% CI: 0.98~1.21, p = 0.13; I^2^ = 33%). Subgroup analyses showed that MetS was independently associated with an increased risk of severe KOA that needed total knee arthroplasty (RR = 1.16, 95% CI: 1.03~1.30, p = 0.02; I^2^ = 0%), but not the risk of total symptomatic KOA (RR = 0.84, 95% CI: 0.65~1.08, p = 0.18; I^2^ = 4%; Fig 3). The heterogeneity within the subgroups were mild, and the difference for the results between the subgroups were significant (p = 0.03), suggesting that the difference of definitions for KOA accounts for the heterogeneity among the studies included. Stratified analyses suggested that MetS was independently associated with an increased risk of KOA in women (RR = 1.23, 95% CI: 1.03~1.47, p = 0.02; I^2^ = 0%), but not in men (RR = 0.90, 95% CI: 0.70~1.14, p = 0.37; I^2^ = 0%; Fig 4). The difference between stratums by sex was also significant (p = 0.04).

### Publication bias

**Fig 5:**
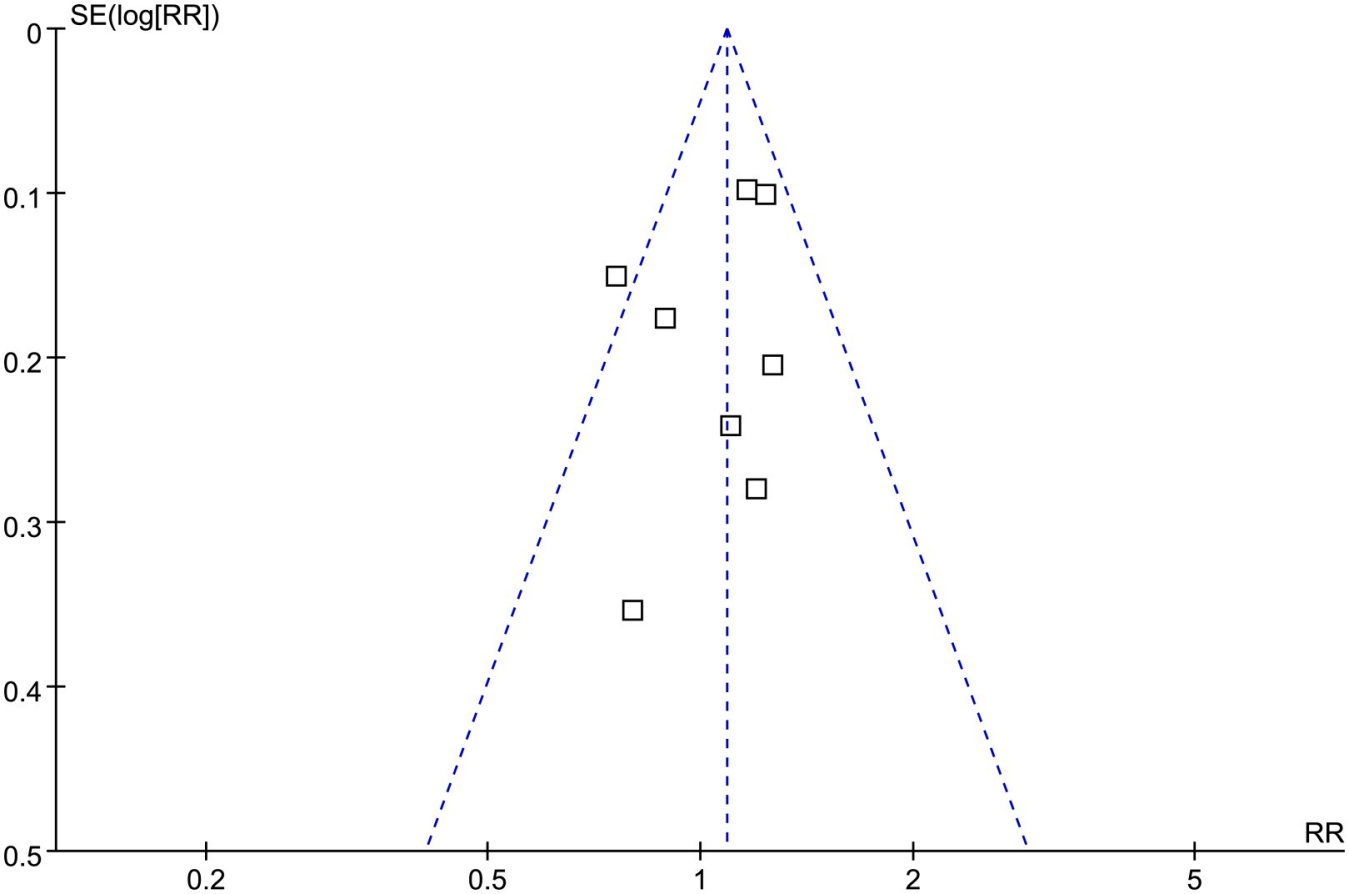
Funnel plots for the meta-analysis of the association between MetS and KOA incidence in general population.

The funnel plots for the association between MetS and incidence of KOA were symmetrical on visual inspection (Fig 5), suggesting low risk of publication bias. Results of Egger’s regression test showed similar results (p = 0.289).

## Discussion

In this meta-analysis, we found that MetS was not associated with a significant risk of overall KOA in general population. However, subgroup analyses indicated that MetS may be associated with an increased risk of severe KOA in general population, but not with an increased risk of overall KOA events. Moreover, a gender-difference of the association between MetS and KOA was suggested by the results of stratified analyses. Specifically, MetS was independently associated with increased incidence of KOA in women but not in men. Taken together, current evidence from prospective cohort studies did not support that MetS was an independent risk factor for overall KOA in general population.

To the best of our knowledge, only one meta-analysis has been published in 2016 that evaluated the association between MetS and KOA [13]. This meta-analysis included seven observational studies (only one prospective cohort study) showed that MetS is significantly associated with higher odds of KOA [13]. However, studies with univariate and multivariate data were both included, which prevented the authors to draw a firm conclusion whether MetS is independently associated with KOA, particularly after considering the potential influence of BMI [13]. Our study, in contrary, only included prospective cohort studies with multivariate adjusted data, showed that MetS is not independently associated with a significant risk for overall KOA in general population, after adjustment of potential confounding factors such as age, sex, BMI, and activity workload. The non-significant association between MetS and KOA risk may be explained by the fact that except for obesity as reflected by BMI, studies evaluating the association between other components of MetS and KOA risk retrieved mixed results. An early meta-analysis of eight observational studies in 2017 confirmed that hypertension was associated with an increased odd of KOA. However, only two of the included studies were cohort studies, and five of them only provided data of univariate analyses [33]. Moreover, a recent meta-analysis with nine studies showed that even though pooled results of case-control and cross-sectional studies suggested a strong relationship between dyslipidemia and KOA, this relationship was not validated by meta-analysis of only cohort studies [34]. Similarly, an updated meta-analysis with 31 studies failed to show that diabetes is an independent risk factor for KOA, after incorporating BMI into the analysis [35]. Taken together, these results indicated that MetS was not an independent risk factor for overall KOA in general population after adjustment of confounding factors including BMI.

Our subgroup analysis suggests that MetS is independently associated with increased incidence of severe KOA that needed TKA, but not associated with the increased incidence of symptomatic KOA or KOA related hospitalization. The difference in the definitions of the KOA outcomes among the included studies explained heterogeneity of the meta-analysis. Although the potential mechanisms underlying these results are unknown, our findings may suggest that MetS is associated with an increased risk of severe KOA. This seemed to be consistent with the findings of previous studies which showed that accumulation of MetS components may be associated with a higher intensity of knee pain [36,37]. Since currently, curative treatments for KOA remain lacking [38], these findings may highlight the hypothesis that optimized management of MetS may be effective to prevent severe KOA in people with MetS. Besides, results of the stratified analyses suggest that MetS may independently predict the risk of KOA in women, but not in men. It has been confirmed that women is associated higher incidence of KOA than men [39]. A previous study in age-matched healthy volunteers in geriatric population demonstrated that the mechanical loading of the female knee is larger than that of the male knee [40]. In addition, the knee cartilage volume was generally smaller in women than that in men [41], and women have increased rates of cartilage loss and progression of cartilage defects at the knee than men [42,43]. Besides, as KOA progressed, both dynamic deformation of lower extremities and degeneration of articular cartilage could be found in females, while no obvious dynamic deformations were found in males [44]. These differences may lead to higher vulnerability of women to severe KOA potentially resulted by metabolic abnormalities and related inflammatory response in MetS, as compared with men. Besides, it could be hypothesized that sex hormone may contribute to the subgroup results that MetS is a risk factor for KOA in women not in men [45]. However, interactions between sex hormones and KOA are complicated and remain to be determined, which warrants future studies. However, results of above subgroup analysis may be of significance for the designing of future studies regarding the association between MetS and KOA.

Our meta-analysis has limitations. Firstly, only five studies were included. Due to the limited number of studies in each subgroup, the results of subgroup and stratified analyses should be interpreted with caution. The findings of subgroup and stratified analyses in this meta-analysis should be treated as hypothesis-generating, which requires further validation in future studies. Secondly, different diagnostic criteria for MetS were applied among the included studies. Although our sensitivity analysis by excluding the study with IDF criteria showed similar results, future studies are needed to determine whether the difference in diagnostic criteria for MetS may affect the association. Thirdly, MetS was evaluated at baseline for all of the included prospective cohort studies. The controlling status of the components of MetS was unknown, which may also affect the outcome of the meta-analysis. In addition, the follow-up durations varied significantly among the included studies (from 6.8 years to 32 years). However, sensitivity analysis by excluding the study with the longest follow-up duration (32 years) [27] showed similar results. Finally, all of the included studies were performed in Western countries and included mainly the Caucasians. The association between MetS and the incidence of KOA in other area, such as in Asia, should be evaluated in prospective cohort studies too.

In conclusion, current evidence from prospective cohort studies did not support MetS was an independent risk factor of overall KOA in general population. However, MetS may be associated with an increased risk of severe KOA in general population, or overall KOA risk in women.

## Supporting information

S1 PRISMA Checklist (DOC)
